# The performance of the Dutch Safety Management System frailty tool to predict the risk of readmission or mortality in older hospitalised cardiac patients

**DOI:** 10.1186/s12877-021-02243-5

**Published:** 2021-05-08

**Authors:** Patricia Jepma, Lotte Verweij, Arno Tijssen, Martijn W. Heymans, Isabelle Flierman, Corine H. M. Latour, Ron J. G. Peters, Wilma J. M. Scholte op Reimer, Bianca M. Buurman, Gerben ter Riet

**Affiliations:** 1 Department of Cardiology, Amsterdam UMC, Amsterdam, the Netherlands; 2 Centre of Expertise Urban Vitality, Faculty of Health, Amsterdam University of Applied Sciences, Amsterdam, the Netherlands; 3 Department of Epidemiology and Data Science, Amsterdam UMC, Amsterdam, the Netherlands; 4 Department of Internal Medicine, section of Geriatric Medicine, Amsterdam UMC, Amsterdam, the Netherlands; 5 Research Group Chronic Diseases, HU University of Applied Sciences Utrecht, Utrecht, the Netherlands

**Keywords:** Aged, Cardiovascular diseases, Frailty, Mortality, Patient readmission, Predictive value of tests, Risk assessment

## Abstract

**Background:**

Early identification of older cardiac patients at high risk of readmission or mortality facilitates targeted deployment of preventive interventions. In the Netherlands, the frailty tool of the Dutch Safety Management System (DSMS-tool) consists of (the risk of) delirium, falling, functional impairment, and malnutrition and is currently used in all older hospitalised patients. However, its predictive performance in older cardiac patients is unknown.

**Aim:**

To estimate the performance of the DSMS-tool alone and combined with other predictors in predicting hospital readmission or mortality within 6 months in acutely hospitalised older cardiac patients.

**Methods:**

An individual patient data meta-analysis was performed on 529 acutely hospitalised cardiac patients ≥70 years from four prospective cohorts. Missing values for predictor and outcome variables were multiply imputed. We explored discrimination and calibration of: (1) the DSMS-tool alone; (2) the four components of the DSMS-tool and adding easily obtainable clinical predictors; (3) the four components of the DSMS-tool and more difficult to obtain predictors. Predictors in model 2 and 3 were selected using backward selection using a threshold of *p* = 0.157. We used shrunk c-statistics, calibration plots, regression slopes and Hosmer-Lemeshow *p*-values (P_HL_) to describe predictive performance in terms of discrimination and calibration.

**Results:**

The population mean age was 82 years, 52% were males and 51% were admitted for heart failure. DSMS-tool was positive in 45% for delirium, 41% for falling, 37% for functional impairments and 29% for malnutrition. The incidence of hospital readmission or mortality gradually increased from 37 to 60% with increasing DSMS scores. Overall, the DSMS-tool discriminated limited (c-statistic 0.61, 95% 0.56–0.66). The final model included the DSMS-tool, diagnosis at admission and Charlson Comorbidity Index and had a c-statistic of 0.69 (95% 0.63–0.73; P_HL_ was 0.658).

**Discussion:**

The DSMS-tool alone has limited capacity to accurately estimate the risk of readmission or mortality in hospitalised older cardiac patients. Adding disease-specific risk factor information to the DSMS-tool resulted in a moderately performing model. To optimise the early identification of older hospitalised cardiac patients at high risk, the combination of geriatric and disease-specific predictors should be further explored.

**Supplementary Information:**

The online version contains supplementary material available at 10.1186/s12877-021-02243-5.

## Background

Hospitalisation of older cardiac patients is associated with increased risk of functional loss, readmission or mortality [1–3]. Geriatric conditions such as malnutrition, tendency to fall and functional impairment are common in older cardiac patients and contribute to these adverse health outcomes [2, 4, 5].

Measurement of risk in older cardiac patients facilitates early initiation of targeted interventions to delay or prevent complications such as (further) functional loss, readmission or mortality in those patients susceptible to such interventions [6]. Risk stratification may help to determine in which patients guideline-recommended treatments may be deployed and for which patients harms outweigh benefits [4, 7, 8].

The Dutch Safety Management System (VeiligheidsManagementSysteem, DSMS) of the Ministry of Health, Welfare and Sport, developed the DSMS-screening tool to detect hospitalised older patients at high risk of functional loss [9]. The DSMS-tool has been in use since 2012 and all Dutch hospitals are required to screen hospitalised older patients on (their risk of) four geriatric domains; delirium, falling, functional impairment and malnutrition. Functional loss is associated with a high risk of readmission and mortality [10–13]. As the DSMS detects frail older patients at high risk of functional loss, the tool may also be capable of identifying patients at high risk of these adverse outcomes and if so, would enable timely targeted deployment of preventive interventions. Therefore, the aim of this study is to estimate the performance of the DSMS-tool alone and combined with other predictors in predicting all-cause unplanned hospital readmission or mortality within 6 months in acutely hospitalised older cardiac patients.

## Methods

An individual patient data meta-analysis was performed on 529 acutely hospitalised cardiac patients ≥70 years from four prospective cohort studies: 1) The Hospital-ADL study [12] examined the development and course of geriatric conditions during and after hospitalisation; 2) the Surprise Question Cohort [14] examined to what extent a negative answer of healthcare professionals to the question “would I be surprised if this patient died in the next year?”, corresponded to mortality within the next year; 3) the Transitional Care Bridge study [15], a multi-centre randomised trial (RCT) on nurse-coordinated transitional care. Only patients of the control group were included in this study because the intervention was found to have a statistically significant effect on mortality; 4) the Cardiac Care Bridge [16], a multi-centre RCT. All patients were included in the current study because the interventions proved to be ineffective.

Patients were eligible for the current study if they 1) had been admitted with a cardiac disease, 2) had been acutely hospitalised for ≥48 h, and 3) were aged ≥70 years.

### The DSMS-screening tool

Table 1 shows the content of the DSMS-tool [9]. The tool consists of single yes/no questions that assess the four geriatric conditions to identify patients at high risk of functional loss. The answers to the questions can also be added up to form the total score. Based on the number of geriatric conditions, the DSMS-score therefore ranges between 0 and 4.

**Table 1:** Screening tool for vulnerable elderly of the Dutch Safety Management System

Domain	Instrument	Questions	Cut-off	Score
Delirium risk	Single questions	Assessing whether: 1) the patient has memory problems; 2) the patient needed help with self-care in the last 24 h; 3) the patient has previously had a delirium	> 1 point	1
Fall risk	Single question	Have you fallen in the last 6 months?	yes	1
Functional impairment	KATZ-6 [17]	Assessing whether the patient currently needs help with 1) bathing, 2) dressing, 3) toileting, 4) transferring from bed to a chair, 5) eating, and 6) whether the patient uses incontinence material	> 2 points	1
Malnutrition	SNAQ [18]	Assessing whether the patient: 1) lost weight unintentionally in the last month (> 3 kg) or last 6 months (> 6 kg) and/or 2) has poor appetite in the last month and 3) used supplemental drinks or tube feeding in the last month.	Question 1 = yes and/or question 2 + 3 = yes	1
Total score				0–4

*KATZ-6* [17] Modified KATZ-6 index, *kg* kilogram, *SNAQ* [18] Short Nutritional Assessment Questionnaire

### Outcome

The primary outcome was the performance of the DSMS-tool in predicting six-month all-cause unplanned readmission or mortality. Readmission data were collected from medical files in the participating hospitals and supplemented with patients’ and family members’ self-reported readmissions in other hospitals. Mortality was registered within the original cohorts and originates from medical files, the Dutch National Personal Records Database [19], or information from family members at follow-up.

### Statistical analyses

#### Missing data

Additional file 1 shows the frequency of missing data in the four cohorts. Missing values for predictor and outcome variables were imputed 20 times using the MICE package in R-Studio (version 3.6.1), involving 19 variables, including 3 indicator variables to identify the 4 cohorts [20]. The only continuous variable with missing values, length of stay (days), was log-transformed before imputation. We used predictive mean matching throughout. The complete datasets (m = 20) were analysed separately and the results pooled using the pooled sampling variance method [21].

#### Descriptive statistics

Descriptive statistics are reported as means with standard deviation (SD) for normally distributed continuous variables and medians with interquartile range (IQR) for non-normally distributed data. Categorical variables are reported as frequencies and percentages. The incidence of all-cause unplanned readmission or mortality at 6 months is reported per DSMS-score. DSMS-scores 3 and 4 were merged to indicate high-risk patients due to the limited numbers with score 4.

#### Regression models

The prediction model for readmission or mortality within 6 months was developed and tested by using an individual patient data meta-analysis of prediction models. Both geriatric and disease-specific candidate predictors associated with readmission or mortality were selected. We explored discrimination and calibration of: 1) DSMS alone (delirium, falling, functional impairment and malnutrition); 2) clinical candidate predictors easily obtainable from medical files or by short questions: age, sex, educational level, living arrangement, polypharmacy (≥ 5 medicines), admission in the previous 6 months and cardiac diagnosis at admission, first without and then including the items of the DSMS; 3) a model based on the four components of the DSMS and more difficult to obtain candidate predictors: Charlson comorbidity index, Mini-Mental State Examination (MMSE), handgrip strength, Short Physical Performance Battery (SPPB) and Geriatric Depression Scale-15 and forcing the DSMS-items into the model. In steps 2 and 3, a backward selection procedure was performed. Predictors were retained in the model if their *p*-value was < 0.157, corresponding with Akaike’s information criterion [22]. No dummy variables were included for the included cohorts. Subsequently, we tested the differences in c-statistics between the models by using the D3 method for pooling Likelihood ratio statistics [23]. The DeLong method was used to obtain the standard error of each imputed dataset and subsequently, the difference in pooled c-statistic was calculated [24].

We internally validated the models using 250 bootstrap samples, which were drawn from the original dataset with missing values and missing values filled in by multiple imputation (m = 20) in every single bootstrap sample. We used shrunk c-statistics, calibration plots (Fig. 4, Additional files 2, 3 and 4), regression slopes and Hosmer-Lemeshow *p*-values (P_HL_) to describe discrimination and calibration. Regression coefficients were shrunk by a single shrinkage factor to reduce over-optimism of model performance in new populations [25]. Since two of the data sets were from randomised trials, that used frailty instruments as an inclusion criterion, we tested model calibration on the combined data of the two observational cohorts to ensure application to a more natural target population. We used the psfmi package in R-studio (version 3.6.1) for these analyses. The psfmi package is fully described elsewhere [24].

## Results

### Population characteristics

**Fig. 1:**
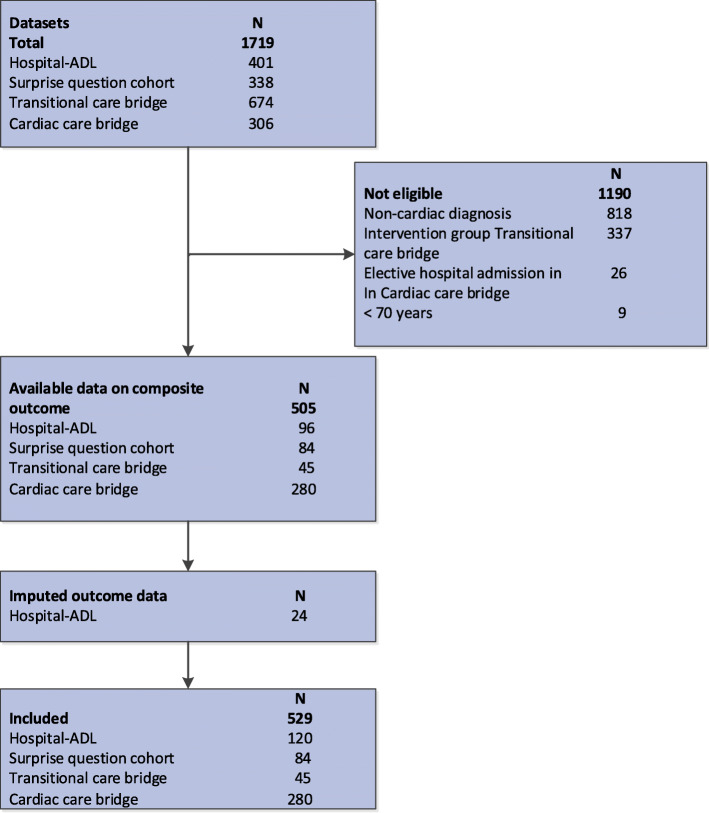
Flowchart

**Fig. 2:**
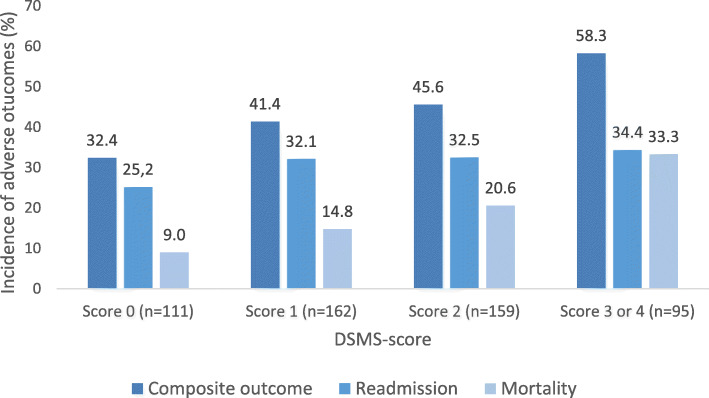
Incidence of adverse outcomes at 6 months follow-up

In total, 529 patients were included in this study (Fig. 1, Table 2). The mean age was 82 years and 52% were males. Most patients had been admitted for heart failure (51%), 38% had been admitted to the hospital in the previous 6 months and 25% of the included patients had cognitive impairment (MMSE < 24). Regarding the DSMS-score, a positive screening was observed in 45% for the risk of delirium, 41% for fall risk, 37% for functional impairment and 29% for malnutrition. The prevalence’s were 21, 31, 30 and, 19% for a DSMS-score of 0, 1, 2 and 3 or 4, respectively. Figure 2 shows the crude incidence of the composite outcome and readmission and mortality separately at 6 months follow-up. The crude incidences of the composite outcome at 6 months were 32, 41, 46 and 58% in patients with DSMS score 0, 1, 2 and 3 or 4, respectively.

**Table 2:** Baseline characteristics

		Hospital-ADL (*n* = 120)	Surprise question cohort (*n* = 84)	Transitional care bridge study (*n* = 45)	Cardiac care bridge (*n* = 280)
**Sociodemographics**					
Age		79.3 ± 6.1	82.8 ± 6.4	81.8 ± 7.6	82.3 ± 6.3
*70–79 years*		65 (45.2)	28 (33.3)	22 (48.9)	86 (30.7)
≥ *80 years*		55 (45.8)	56 (66.7)	23 (51.1)	194 (69.3)
Sex	Female	65 (54.2)	45 (54.8)	17 (37.8)	145 (51.8)
Educational level^a^	Primary school or less	31 (25.8)	35 (40.5)	37 (82.2)	112 (40.0)
	Secondary education	68 (56.6)	34 (40.5)	5 (11.1)	92 (32.9)
	College or university	21 (17.5)	16 (19.0)	3 (6.7)	75 (26.8)
Living arrangement	Living alone	48 (40.0)	44 (52.4)	16 (35.6)	160 (57.1)
**Hospital admission**					
Diagnosis on admission	Heart failure	48 (40.0)	26 (31.0)	25 (55.6)	173 (61.8)
	Acute coronary syndrome	28 (23.3)	33 (39.3)	10 (22.2)	42 (15.0)
	Other	44 (36.7)	25 (29.8)	10 (22.2)	65 (23.2)
Length of stay	Days	5.1 [3.3–8.5]	7.0 [4.0–12.0]	8.0 [5.0–16.5]	7.0 [4.3–10.0]
Hospital admission ≤ 6 months prior to index event		37 (30.8)	20 (23.8)	17 (37.8)	128 (45.7)
**Geriatric conditions**					
Polypharmacy	≥ 5 medicines	79 (65.8)	62 (73.8)	40 (88.9)	225 (80.4)
Charlson Comorbidity Index		1 [1–3]	2 [1–4]	4 [2–5]	3 [1–4]
MMSE		26.5 ± 2.9	25.3 ± 1.8	25.7 ± 3.6	24.7 ± 3.6
Depression	GDS-15	3.4 ± 2.5	4.7 ± 1.5	4.7 ± 1.6	3.4 ± 2.5
Handgrip strength^b^	kg	27.6 ± 10.4	23.7 ± 2.4	18.4 ± 7.3	21.4 ± 8.8
Functional status	SPPB	7.0 ± 3.5	5.5 ± 2.1	5.4 ± 1.8	4.8 ± 2.8
**DSMS-items**^**c**^					
Delirium risk score	DSMS at risk of delirium	19 (15.8)	24 (28.6)	37 (82.2)	159 (56.8)
Fall ≤ 6 months	DSMS risk of falling	39 (32.5)	21 (25.0)	21 (46.7)	133 (47.5)
Functional impairment (KATZ-6)	DSMS impairment in ADL	38 (31.7)	22 (26.2)	23 (51.1)	112 (40.0)
Malnutrition risk (SNAQ)	DSMS risk of malnutrition	32 (26.7)	5 (6.0)	21 (46.7)	94 (33.6)
DSMS score 0		43 (35.8)	44 (52.4)	3 (6.7)	21 (7.5)
DSMS score 1		42 (35.0)	15 (17.9)	8 (17.8)	97 (34.6)
DSMS score 2		24 (20.0)	20 (23.8)	19 (42.2)	97 (34.6)
DSMS score 3 or 4		11 (9.2)	5 (6.0)	15 (33.3)	65 (23.2)

Mean ± standard deviation, median [25–75 centile], N (%). ^a^Primary education: elementary or primary school. Secondary education: pre-vocational, senior general or pre-university. Higher education: higher professional or university, ^b^Dominant hand highest value, ^c^Dutch Safety Management System [9]: the score between 0 and 4 points, based on four domains of frailty: (risk of) delirium, falling, functional impairment, and malnutrition

*Abbreviations*: *ADL* Activities of Daily Living, *DSMS* Dutch Safety and Management System, *GDS* Geriatric Depression Scale, *KATZ-6* [17] Modified KATZ-6 index, *kg* kilogram, *MMSE* Mini-Mental State Examination, *SNAQ* [18] Short Nutritional Assessment Questionnaire, *SPPB* Short Physical Performance Battery

### Performance of the DSMS-tool

**Fig. 3:**
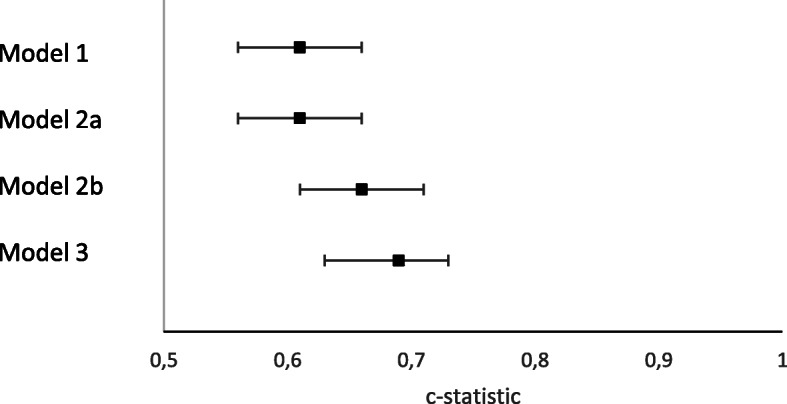
Areas under the curve and 95% confidence intervals for predictors of six-month readmission or mortality. Model 1: DSMS delirium, DSMS fall risk, DSMS functional impairment, DSMS malnutrition. Model 2a: sex, admission in the previous 6 months and cardiovascular diagnosis. Model 2b: sex, admission in the previous 6 months and cardiovascular diagnosis + model 1. Model 3: Charlson comorbidity index [26], cardiovascular diagnosis + model 1

Table 3 and Fig. 3 show the predictive performance of the three models in predicting readmission or mortality within 6 months. In model 1, including the DSMS only, malnutrition was the strongest predictor (OR 2.29, 95% CI 1.47–3.56). The model discriminated limited (c-statistic 0.61, 95% CI 0.56–0.66) and after internal validation discrimination decreased (c-statistic 0.55).

**Table 3:** Multivariable analyses and predictive performance for readmission or mortality at six-months^a^

	Model 1			Model 2a			Model 2b			Model 3		
	OR	95% CI	*p*-value	OR	95% CI	*p*-value	OR	95% CI	*p*-value	OR	95% CI	*p*-value
**DSMS**												
Delirium	1.39	(1.29–1.50)	< 0.001				1.29	(0.93–1.79)	0.127	1.06	(0.76–1.46)	0.740
Fall risk	1.09	(0.77–1.55)	0.642				1.1	(0.81–1.49)	0.551	1.07	(0.80–1.44)	0.664
Functional impairment	1.24	(0.91–1.69)	0.174				1.23	(0.88–1.74)	0.236	1.18	(0.77–1.81)	0.457
Malnutrition	2.21	(1.45–3.38)	< 0.001				1.89	(1.31–2.72)	< 0.001	1.79	(1.26–2.53)	0.001
Female				0.80	(0.61–1.06)	0.113	0.73	(0.54–1.00)	0.045			
Admission previous 6 months				1.33	(0.97–2.13)	0.156	1.34	(0.97–1.84)	0.073			
**Admission diagnosis**												
Heart failure				Reference		0.004	Reference		0.026	Reference		0.102
Acute coronary syndrome				0.74	(0.52–1.06)		0.84	(0.56–1.24)		0.90	(0.62–1.31)	
Other				0.57	(0.40–0.79)		0.60	(0.42–0.87)		0.68	(0.48–0.97)	
**Charlson comorbidity Index**												
Score 0										Reference		0.002
Score 1										1.12	(0.64–1.96)	
Score 2										1.06	(0.59–1.90)	
Score 3										1.71	(0.95–3.07)	
Score 4										1.93	(1.02–3.66)	
Score ≥ 5										2.72	(1.42–5.27)	

Model 1: DSMS delirium, DSMS fall risk, DSMS functional impairment, DSMS malnutrition

Model 2a: sex, admission in the previous 6 months and cardiovascular diagnosis

Model 2b: sex, admission in the previous 6 months and cardiovascular diagnosis + model 1

Model 3: Charlson comorbidity index [26], cardiovascular diagnosis + model 1

*Abbreviations*: *DSMS* Dutch Safety Management System

^a^No dummy variables for the four cohorts were included in the multivariable analyses

In model 2a (without the DSMS-items) only sex, admission in the previous 6 months and diagnosis at admission remained in the model. In model 2b, the DSMS-items were added to the predictors in 2a which slightly improved discrimination (c-statistic 0.66, 95% CI 0.61–0.71). The discrimination of model 2b was statistically significantly better than that of model 1 (*p* = 0.002). In the observational cohorts, the c-statistic of model 2b was 0.57 (95% CI 0.48–0.65), however, the model was well calibrated (corrected slope 0.71, P_HL_ = 0.89) (Additional files 2 and 3).

**Fig. 4:**
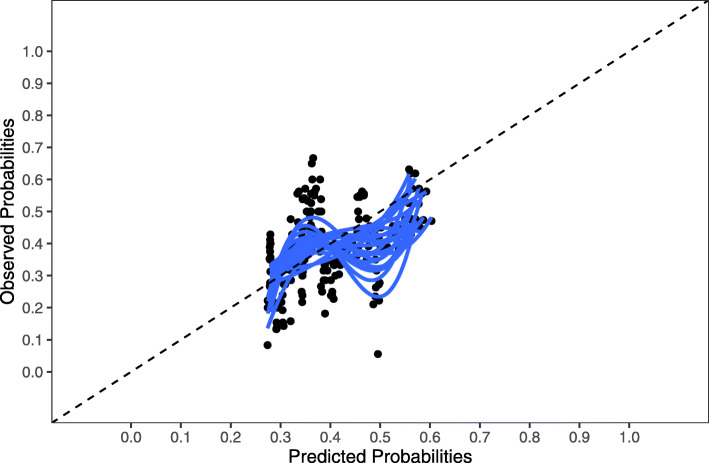
Calibration plot of readmission or mortality within 6 months (model 3) in the two observational cohorts

In model 3, the admission diagnosis and Charlson comorbidity index were selected, which yielded a model c-statistic of 0.69 (95% CI 0.63–0.73), which fell to 0.66 after internal validation. Model 3 discriminated statistically significantly better than model 1 (*p* < 0.001) and model 2b (*p* = 0.007). The calibration plot of model 3 is shown in Additional file 4. In the observational cohorts, the discriminative performance was lower (c-statistic 0.58, 95% CI 0.47–0.68) but well calibrated (corrected slope 0.76, P_HL_ = 0.66) as shown in Fig. 4.

## Discussion

We examined the performance of the DSMS-tool, alone and combined with other predictors, on all-cause unplanned hospital readmission or mortality within 6 months in older patients acutely hospitalised for a cardiac reason. Our results show that the DSMS-tool’s performance is limited in this population. However, in combination with the diagnosis on admission and the Charlson comorbidity index, reasonably good predictions could be made.

Originally, the DSMS-items were introduced into Dutch hospitals to assess the risk of functional loss in older patients on admission and to selectively deploy interventions to prevent functional loss early [9]. However, the predictive performance has not been studied before implementation in 2012. Heim et al. [27] studied discrimination of the DSMS-tool in predicting the occurrence of a composite outcome of death, high healthcare demand or at least one additional dependency in activities of daily living within 3 months follow-up among acutely and electively hospitalised patients ≥70 years at departments of neurology, urology, surgery and orthopaedics. On external validation in 812 patients (of which 105 only had data on healthcare demand), they found a sensitivity of 0.61 and a specificity of 0.75 (c-statistics 0.68) for the DSMS-tool reinforced by information on age (cut-off at 80 years). Using different methods (cardiac patients, all acutely admitted, six-month composite outcome of readmission or death, multiple imputation of missing values, bootstrapping and shrinkage), we found that discrimination of the DSMS-tool to predict the occurrence of six-month hospital readmission or mortality was much lower (shrunk c-statistic = 0.55). Although the contrasting c-statistics may be explained by the different outcome measures and time window, it could also be explained by differences between the study populations. For example, Heim et al. [27] included both acutely and electively hospitalised patients including a high percentage of surgical and orthopaedic patients, whereas we focussed solely on the acutely hospitalised cardiac population in which a high prevalence of geriatric conditions and comorbidities were found. In addition, more patients in our study were cognitively impaired (MMSE ≤23 21.3% versus 15.9%) [27]. Surprisingly, and despite a fairly wide range of ages in our study, age was not a strong predictor and was not selected in any of the models.

Hermans et al. [28] studied, in a retrospective analysis of routine data, the association between the DSMS-score and the occurrence of mortality or a composite of various complications after a percutaneous coronary intervention within 30 days in patients with ST-elevated myocardial infarction ≥70 years. They found an OR of 9.6 (95%CI 1.6–56.9) for a DSMS-score (≥ 1) to predict 30-day mortality. However, the authors were hindered by the low incidence of mortality (*n* = 11; 5%) which may have led to severe overfitting of their regression model.

Until now, only few studies have studied the performance of the DSMS-tool. These studies vary in study population, time window, outcomes and methods and are therefore difficult to compare. As a result, more research is needed to study the performance of the DSMS-tool, especially since in the Netherlands its use is compulsory in all patients ≥70 years who are hospitalised. In our analyses, we focussed on the original and routinely used, binary cut-off points within the four domains of the DSMS. For further research, it would be interesting to elucidate the performance of the most efficient subset of these 13 items within the four domains, possibly modeling these as a continuous score.

In addition, it is important to not only identify patients at risk but also act on it, that is, initiate early preventive interventions in those patients indicated by their predicted high risk. As far as we are aware, treatment thresholds, in terms of predicted risk, are seldom specified. Within the DSMS-tool, attention is payed to practical hospital-based interdisciplinary interventions in patients with one or more risk factors present [9]. However, it is known that common geriatric syndromes are often still present 3 months post-discharge [12]. The DSMS recommends transferring risk information to caregivers in primary care. However, more attention may be needed to continue interventions from hospital to home. For example, transitional care interventions contribute to continuity of care across care settings and have been shown to reduce the risk of readmission and mortality in several populations [29, 30].

We conclude that a combination of variables reflecting geriatric conditions (the DSMS-items and the Charlson comorbidity index) and a disease-related factor (diagnosis at admission), led to better predictive performance than a model of the DSMS-items alone. A recent systematic review of risk prediction models in cardiac patients showed that only few studies use geriatric predictors, such as physical performance or dementia, to estimate patients’ probabilities of experiencing an unplanned readmission (van Grootven, submitted). However, models containing geriatric predictors did not seem to predict differently than those without. This may partly be explained by the relatively low mean age in the underlying studies as most studies included patients ≤70 years. This lowers the presence of geriatric syndromes, which may hinder accurate detection of potential predictive capabilities. The SILVER-AMI study included patients ≥75 years and developed risk prediction models for 30 and 180-day readmission [2, 31]. In accordance with our results, they found that a combination of geriatric as well as disease-specific risk factors best predicted the risk of readmission.

### Strengths and limitations

In this study we combined data of older cardiac patients of four studies to examine the performance of the DSMS-tool and the contribution of additional variables using rigorous statistical methods. Our study contributes to the evidence on how to identify older cardiac patients at risk of readmission or mortality.

Some limitations should however be considered. First, we examined the performance of the DSMS-tool on the risk estimation of hospital readmission or mortality in older cardiac patients. However, the tool has originally been developed to identify older patients at risk of functional loss. Since functional loss is strongly related to hospital readmission or mortality, testing the performance of the DSMS-tool on these outcomes is considered plausible [10, 11]. Second, no c-statistics for readmission and mortality as separate outcomes were reported due to the limited number of events per outcome. However, the outcome-specific preventive options to be considered after high-probability predictions of readmission or death from a model are comparable [32]. Third, while we were able to select a broad range of geriatric predictors, some important medical (disease-specific) predictors (e.g. left ventricular ejection fraction, and stage of disease (NYHA)) were not available. Information on these tests is usually not available on hospital admission (and in our four cohorts) and were therefore not included in our model which focusses on the early admission phase. However, data about the disease history and comorbidities may be available at hospital admission. For example, the presence of specific comorbidities such as renal failure, diabetes [33, 34] or chronic obstructive pulmonary disease [2, 31] are known to increase the risk of adverse outcomes and may be of additional value in future risk prediction models for older cardiac patients. Fourth, in the two intervention cohorts a selected subgroup of 87% frail older cardiac patients according to the DSMS-tool was included, compared to 44% in the two observational cohorts. We therefore performed a second internal validation process on the two observational cohorts to reflect model performance in a hospitalised older cardiac patient population representative of that encountered in clinical practice. Last, despite rigorous steps taken to assess the internal validity of our models, an additional external validation in independent datasets is recommended to examine the generalisability of our results.

## Conclusion

The DSMS-tool alone has limited capacity to accurately estimate the risk of readmission or mortality in hospitalised older cardiac patients. Adding disease-specific risk factor information to the DSMS-tool resulted in a moderately performing model. To optimise the early identification of older hospitalised cardiac patients at risk, the combination of geriatric and disease-specific predictors should be further explored.

## Supplementary Information

**Additional file 1.** Frequency of missing data per variable in the four cohorts.

**Additional file 2: Supplemental Figure 1.** Calibration plot of readmission or mortality within 6 months (model 2b) in 250 bootstrapped samples.

**Additional file 3: Supplemental Figure 2.** Calibration plot of readmission or mortality within 6 months (model 2b) in the two observational cohorts.

**Additional file 4: Supplemental Figure 3.** Calibration plot of readmission or mortality within 6 months (model 3), in 250 bootstrapped samples.

## Data Availability

The datasets used and/or analysed during the current study are available from the corresponding author on reasonable request.

## Abbreviations

CI: Confidence interval
DSMS: Dutch Safety Management System
HL: Hosmer-Lemeshow
IQR: Interquartile range
MMSE: Mini-Mental State Examination
OR: Odds ratio
RCT: Randomised controlled trial
SD: Standard deviation
SPPB: Short Physical Performance Battery

## References

[1] Khera R, Wang Y, Bernheim SM, Lin Z, Krumholz HM (2020). Post-discharge acute care and outcomes following readmission reduction initiatives: national retrospective cohort study of Medicare beneficiaries in the United States. BMJ.

[2] Dodson JA, Hajduk AM, Murphy TE, Geda M, Krumholz HM, Tsang S (2019). Thirty-day readmission risk model for older adults hospitalized with acute myocardial infarction. Circ Cardiovasc Qual Outcomes.

[3] Jepma P, Ter Riet G, van Rijn M, Latour CHM, Peters RJG, WJM SOR (2019). Readmission and mortality in patients >/=70 years with acute myocardial infarction or heart failure in the Netherlands: a retrospective cohort study of incidences and changes in risk factors over time. Neth Heart J.

[4] Gorodeski EZ, Goyal P, Hummel SL, Krishnaswami A, Goodlin SJ, Hart LL, Forman DE, Wenger NK, Kirkpatrick JN, Alexander KP, Geriatric Cardiology Section Leadership Council, American College of Cardiology (2018). Domain management approach to heart failure in the geriatric patient: present and future. J Am Coll Cardiol.

[5] Bell SP, Orr NM, Dodson JA, Rich MW, Wenger NK, Blum K, Harold JG, Tinetti ME, Maurer MS, Forman DE (2015). What to expect from the evolving field of geriatric cardiology. J Am Coll Cardiol.

[6] Vitale C, Jankowska E, Hill L, Piepoli M, Doehner W, Anker SD, Lainscak M, Jaarsma T, Ponikowski P, Rosano GMC, Seferovic P, Coats AJ (2019). Heart Failure Association/European Society of Cardiology position paper on frailty in patients with heart failure. Eur J Heart Fail.

[7] Boyd C, Smith CD, Masoudi FA, Blaum CS, Dodson JA, Green AR, Kelley A, Matlock D, Ouellet J, Rich MW, Schoenborn NL, Tinetti ME (2019). Decision making for older adults with multiple chronic conditions: executive summary for the American Geriatrics Society guiding principles on the care of older adults with multimorbidity. J Am Geriatr Soc.

[8] Zão A, Magalhães S, Santos M (2019). Frailty in cardiovascular disease: screening tools. Rev Port Cardiol.

[9] Dutch Safety Management Program. Practical guide for frail older patients [in Dutch]. Place unknown: Dutch Safety Management Program; 2009.

[10] van Seben R, Covinsky KE, Reichardt LA, Aarden JJ, van der Schaaf M, van der Esch M, Engelbert RHH, Twisk JWR, Bosch JA, Buurman BM (2020). Insight into the posthospital syndrome: a 3-month longitudinal follow up on geriatric syndromes and their association with functional decline, readmission, and mortality. J Gerontol A Biol Sci Med Sci.

[11] Koch D, Kutz A, Haubitz S, Baechli C, Gregoriano C, Conca A, Volken T, Schuetz P, Mueller B (2020). Association of functional status and hospital-acquired functional decline with 30-day outcomes in medical inpatients: a prospective cohort study. Appl Nurs Res.

[12] van Seben R, Reichardt LA, Aarden JJ, van der Schaaf M, van der Esch M, Engelbert RHH (2019). the course of geriatric syndromes in acutely hospitalized older adults: the hospital-ADL Study. J Am Med Dir Assoc.

[13] Buurman BM, Hoogerduijn JG, van Gemert EA, de Haan RJ, Schuurmans MJ, de Rooij SE (2012). Clinical characteristics and outcomes of hospitalized older patients with distinct risk profiles for functional decline: a prospective cohort study. PLoS One.

[14] Flierman I, van Rijn M, Willems DL, Buurman BM (2020). Usability of the surprise question by nurses to identify 12-month mortality in hospitalized older patients: a prospective cohort study. Int J Nurse Stud.

[15] Buurman B, Parlevliet J, Allore H, Blok W, van Deelen B, Moll van Charante E (2016). Comprehensive geriatric assessment and transitional care in acutely hospitalized patients - the transitional care bridge randomized clinical trial. JAMA Intern Med.

[16] Verweij L, Jepma P, Buurman BM, Latour CHM, Engelbert RHH, Ter Riet G (2018). The cardiac care bridge program: design of a randomized trial of nurse-coordinated transitional care in older hospitalized cardiac patients at high risk of readmission and mortality. BMC Health Serv Res.

[17] Katz S, Ford A, Moskowitz R, Jackson B, Jaffe M (1963). Studies of illness in the aged. The index of Adl: a standardized measure of biological and psychosocial function. JAMA.

[18] Kruizenga HM, Seidell JC, de Vet HC, Wierdsma NJ, Van Bokhorst-de van der Schueren MA (2005). Development and validation of a hospital screening tool for malnutrition: the short nutritional assessment questionnaire (SNAQ). Clin Nutr.

[19] Government of the Netherlands. What information is in the Personal Records Database? Available at: https://www.government.nl/topics/personal-data/question-and-answer/what-information-is-in-the-personal-records-database. Accessed 02 Jul 2020.

[20] Held U, Kessels A, Garcia Aymerich J, Basagaña X, Ter Riet G, Moons KG (2016). Methods for handling missing variables in risk prediction models. Am J Epidemiol.

[21] Heymans M, Eekhout I. Chapter 13. Pooling methods for categorical variables. Available at: https://bookdown.org/mwheymans/bookmi/pooling-methods-for-categorical-variables.html. Accessed 04 Feb 2021.

[22] Sauerbrei W (1999). The use of resampling methods to simplify regression models in medical statistics. J R Stat Soc Ser.

[23] Meng X, Rubin D (1992). Performing likelihood ratio tests with multiply-imputed data sets. Biometrica.

[24] Heymans M. Psfmi package. Available at: https://mwheymans.github.io/psfmi/index.html. Accessed 20 Jan 2021.

[25] Van Houwelingen JC, Le Cessie S (1990). Predictive value of statistical models. Stat Med.

[26] Charlson ME, Pompei P, Ales KL, MacKenzie CR (1987). A new method of classifying prognostic comorbidity in longitudinal studies: development and validation. J Chronic Dis.

[27] Heim N, van Fenema EM, Weverling-Rijnsburger AW, Tuijl JP, Jue P, Oleksik AM (2015). Optimal screening for increased risk for adverse outcomes in hospitalised older adults. Age Ageing.

[28] Hermans MPJ, Eindhoven DC, van Winden LAM, de Grooth GJ, Blauw GJ, Muller M, Schalij MJ (2019). Frailty score for elderly patients is associated with short-term clinical outcomes in patients with ST-segment elevated myocardial infarction treated with primary percutaneous coronary intervention. Neth Heart J.

[29] Naylor MD, Shaid EC, Carpenter D, Gass B, Levine C, Li J, Malley A, McCauley K, Nguyen HQ, Watson H, Brock J, Mittman B, Jack B, Mitchell S, Callicoatte B, Schall J, Williams MV (2017). Components of comprehensive and effective transitional care. J Am Geriatr Soc.

[30] Naylor MD, Aiken LH, Kurtzman ET, Olds DM, Hirschman KB (2011). The care span: The importance of transitional care in achieving health reform. Health Aff.

[31] Dodson JA, Hajduk AM, Murphy TE, Geda M, Krumholz HM, Tsang S, Nanna MG, Tinetti ME, Ouellet G, Sybrant D, Gill TM, Chaudhry SI (2021). 180-day readmission risk model for older adults with acute myocardial infarction: the SILVER-AMI study. Open Heart.

[32] Le Berre M, Maimon G, Sourial N, Gueriton M, Vedel I (2017). Impact of transitional care services for chronically ill older patients: a systematic evidence review. J Am Geriatr Soc.

[33] Keenan PS, Normand SL, Lin Z, Drye EE, Bhat KR, Ross JS (2008). An administrative claims measure suitable for profiling hospital performance on the basis of 30-day all-cause readmission rates among patients with heart failure. Circ Cardiovasc Qual Outcomes.

[34] Krumholz HM, Lin Z, Drye EE, Desai MM, Han LF, Rapp MT, Mattera JA, Normand SLT (2011). An administrative claims measure suitable for profiling hospital performance based on 30-day all-cause readmission rates among patients with acute myocardial infarction. Circ Cardiovasc Qual Outcomes.

