# Supplementary material for: The performance of the Dutch Safety Management System frailty tool to predict the risk of readmission or mortality in older hospitalised cardiac patients
Source: BMC Geriatr. 2021 May 8;21:299. doi: 10.1186/s12877-021-02243-5 (PMC8105911; doi:10.1186/s12877-021-02243-5)
Supplement: Supplementary file 1 — Additional file 1. Frequency of missing data per variable in the four cohorts. [file 12877_2021_2243_MOESM1_ESM.docx]

**Additional file 1. Frequency of missing data per variable in the four cohorts**

|  | Hospital-ADL  (n=120) | Surprise question cohort (n=84) | Transitional care bridge study (n=45) | Cardiac care bridge study (n=280) |
| --- | --- | --- | --- | --- |
| **Sociodemographics** |  |  |  |  |
| Age | 0 | 0 | 0 | 0 |
| Gender | 0 | 0 | 0 | 0 |
| Educational level | 0 | 84 | 0 | 1 |
| Living arrangement | 0 | 0 | 0 | 0 |
| **Hospital admission** |  |  |  |  |
| Diagnosis on admission | 0 | 0 | 0 | 0 |
| Length of stay | 4 | 1 | 0 | 0 |
| Hospital admission ≤6 months prior to index event | 0 | 1 | 45 | 0 |
| **Geriatric conditions** |  |  |  |  |
| Polypharmacy | 2 | 3 | 2 | 6 |
| Charlson Comorbidity Score | 0 | 0 | 1 | 0 |
| MMSE | 7 | 84 | 1 | 0 |
| Depression | 2 | 84 | 45 | 2 |
| Handgrip strength | 26 | 84 | 21 | 33 |
| Functional status | 36 | 84 | 45 | 92 |
| **DSMS-items** |  |  |  |  |
| Delirium risk score | 0 | 5 | 1 | 0 |
| Activities of Daily Living (KATZ-6) | 0 | 2 | 0 | 0 |
| Malnutrition risk (SNAQ) | 1 | 2 | 2 | 0 |
| Fall ≤6 months | 0 | 6 | 1 | 0 |
| **Outcome** |  |  |  |  |
| Composite outcome on 6 months | 24 | 0 | 0 | 0 |
