# Supplementary figures and images for: The performance of the Dutch Safety Management System frailty tool to predict the risk of readmission or mortality in older hospitalised cardiac patients
Source: BMC Geriatr. 2021 May 8;21:299. doi: 10.1186/s12877-021-02243-5 (PMC8105911; doi:10.1186/s12877-021-02243-5)

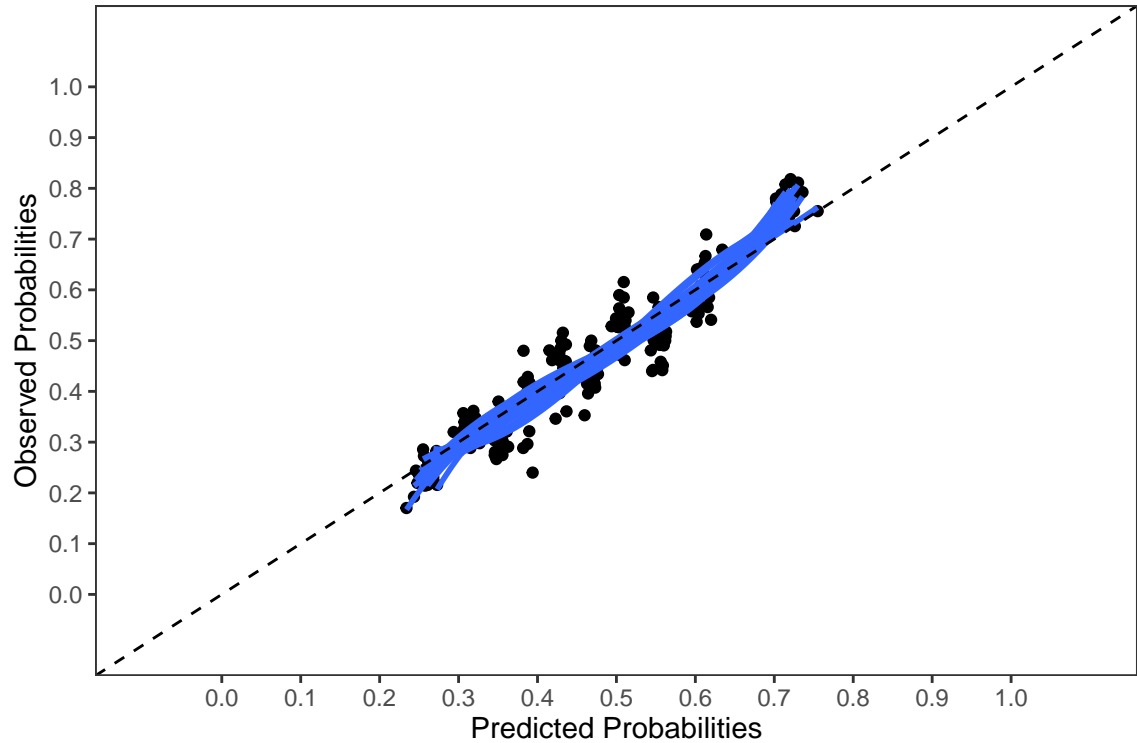

Supplement: Supplementary file 2 — Additional file 2: Supplemental Figure 1. Calibration plot of readmission or mortality within 6 months (model 2b) in 250 bootstrapped samples. [file 12877_2021_2243_MOESM2_ESM.pdf]

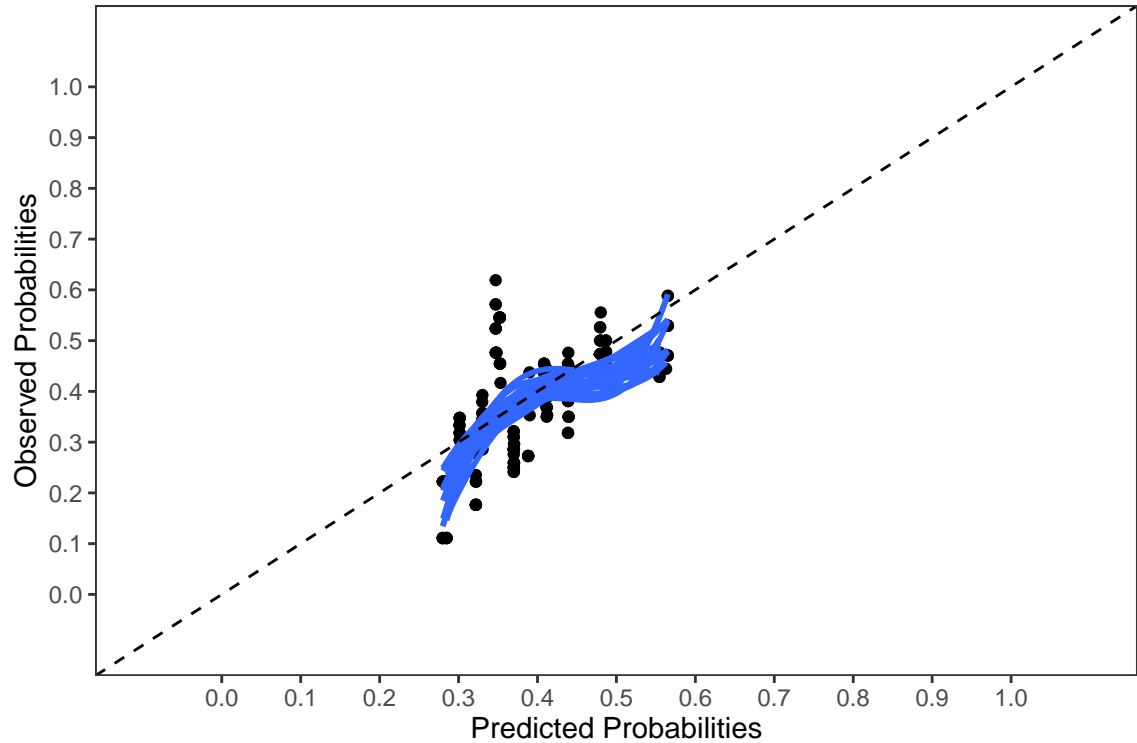

Supplement: Supplementary file 3 — Additional file 3: Supplemental Figure 2. Calibration plot of readmission or mortality within 6 months (model 2b) in the two observational cohorts. [file 12877_2021_2243_MOESM3_ESM.pdf]

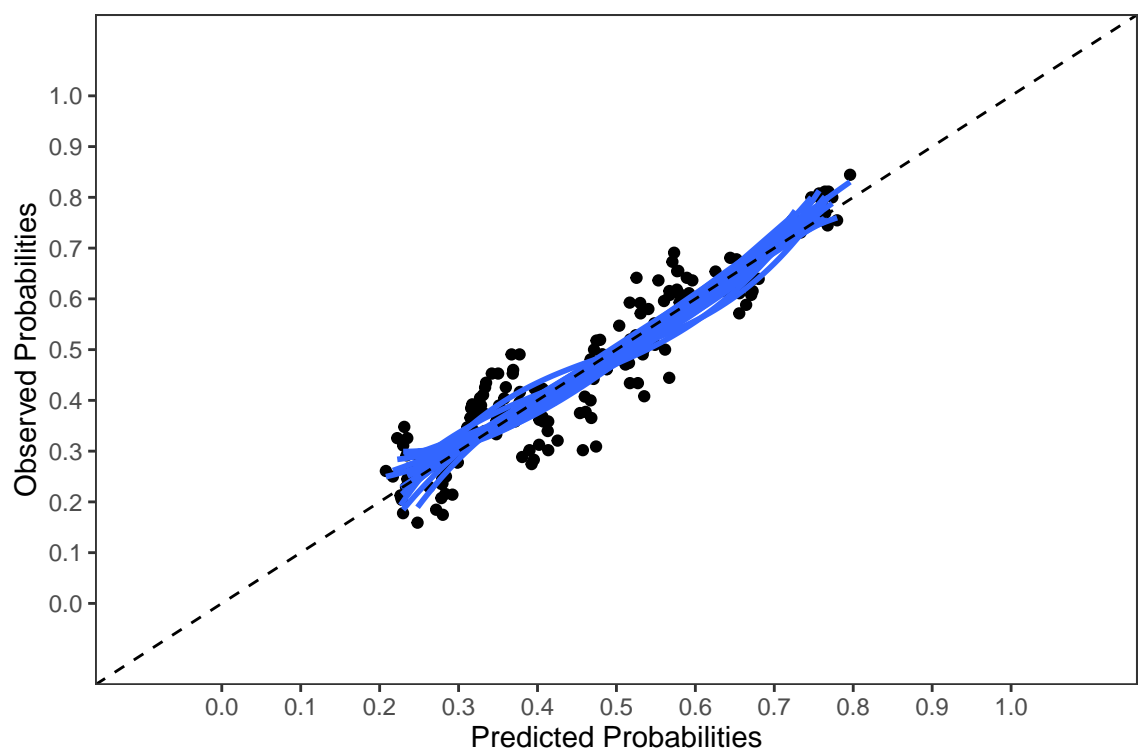

Supplement: Supplementary file 4 — Additional file 4: Supplemental Figure 3. Calibration plot of readmission or mortality within 6 months (model 3), in 250 bootstrapped samples. [file 12877_2021_2243_MOESM4_ESM.pdf]
